# The effects of rituximab on serum IgE and BAFF

**DOI:** 10.1186/1710-1492-9-39

**Published:** 2013-10-03

**Authors:** Angira Dasgupta, Katherine Radford, Donald M Arnold, Lehana Thabane, Parameswaran Nair

**Affiliations:** 1 Divisions of Respirology, McMaster University, Hamilton, ON, Canada; 2 Divisions of Hematology, McMaster University, Hamilton, ON, Canada; 3 Department of Medicine, McMaster University, Hamilton, ON, Canada; 4 Department of Clinical Epidemiology and Biostatistics, McMaster University, Hamilton, ON, Canada

**Keywords:** Anti-CD20, Rituximab, Immunoglobulin E, BAFF, Asthma

## Abstract

**Background:**

There are few treatment options for patients with severe atopic asthma. Antagonism of IgE is an effective strategy. We investigated, by utilizing serum samples from a clinical trial of Rituximab in patients with Idiopathic Thrombocytopenic Purpura, if B cell depletion would decrease serum IgE and therefore be a potential therapeutic option.

**Findings:**

In a placebo-controlled randomized clinical trial of Rituximab, an anti-CD20 molecule, there were no significant differences in serum levels of IgE or BAFF levels between the two treatment groups at 3 or 6 months irrespective of the baseline serum IgE levels.

**Conclusions:**

Since Rituximab did not significantly decrease serum IgE levels, this proof of concept study suggests that Rituximab may not be a useful treatment strategy for patients with severe IgE mediated disease.

## Findings

The pathogenesis of asthma involves both B and T cell mechanisms. Although not fully understood, B cells and immunoglobulin E (IgE) contribute to the development of atopic asthma, and omalizumab, an anti-IgE monoclonal antibody is approved for use in severe asthma. Another possible B cell target for severe asthma is CD20 which is one of the two candidate genes recently identified in atopy [1]. CD20 is a B cell surface receptor that has a role in B cell proliferation and differentiation. Rituximab is one such chimeric monoclonal anti-CD20 agent which is expected to affect immunoglobulin levels through antibody dependent cytotoxicity and complement dependent cytotoxicity directed against CD20 expressing B cells. It was first developed as a treatment for B cell lymphoma and has subsequently found use in treating certain autoimmune diseases like rheumatoid arthritis, SLE and pemphigous vulgaris, Churg Strauss syndrome including in those patients with asthma [2] and atopic dermatitis where a significant clinical improvement was associated with only a modest reduction in total circulating IgE [3].

Recently another B cell factor called B cell-activating factor (BAFF) that is required to generate and maintain mature B cells has been demonstrated to be elevated in serum and sputum of asthmatics [4,5]. High BAFF levels correlated with airway hyperresponsiveness (AHR) and reduced FEV1 independent of IgE levels. Blocking BAFF activity by inhibitors has led to improvement in AHR in mice [6]. Reports on BAFF levels following rituximab therapy have been inconsistent so far with increased levels being reported in one study [7]. Rituximab may be an attractive therapeutic option for patients with severe allergic asthma if it reduces serum IgE and BAFF.

We tested this hypothesis in a post-hoc analysis of serum obtained from a placebo-controlled randomized clinical trial of rituximab, administered at a dose of 375 mg/m2 for 4 weekly infusions for primary immune thrombocytopenia [8]. This trial offered a unique opportunity to examine the effect of rituximab on IgE and BAFF outside of oncology patients, in whom immunoglobulin levels may be dysregulated because of bone marrow involvement with cancer or toxicities of chemotherapy. Sera from 47 patients (the rest of the 13 samples from the study were not available due to storage problems) were assayed for IgE and BAFF at 3 time points (baseline, end of 3rd month, end of 6th month/end of treatment). The assays were done by quantitative sandwich enzyme immunoassay techniques (R&D Systems, Cedlarlane Corp, Burlington, ON, Canada) and all measurements were done blinded to treatment allocation and clinical response. The values for IgE and BAFF were log transformed and analyzed using ANCOVA with the respective baseline values as covariates.

**Figure 1:**
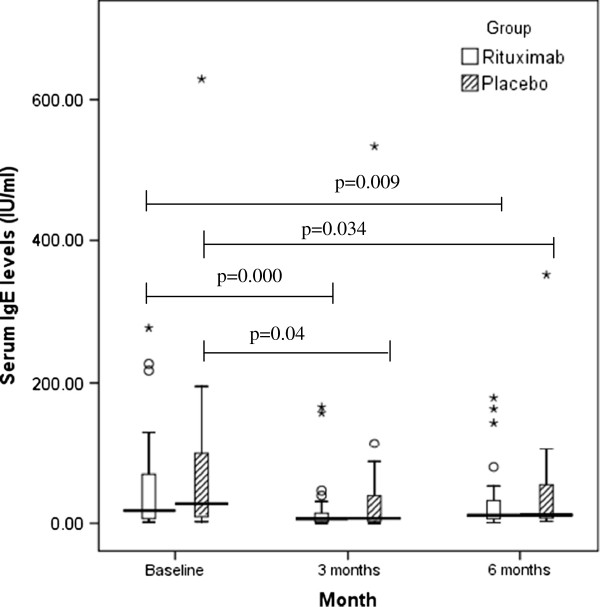
**The effects of Rituximab and Placebo on serum IgE (IU/l).** Although Rituximab significantly reduced IgE levels at 3 and 6 months compared to pre-treatment baseline, this was not significantly different from the effect of placebo treatment at the corresponding time points. (° = outliers with values more than 1.5 times the height of the box; * = outliers with values more than 3 times height of box).

**Figure 2:**
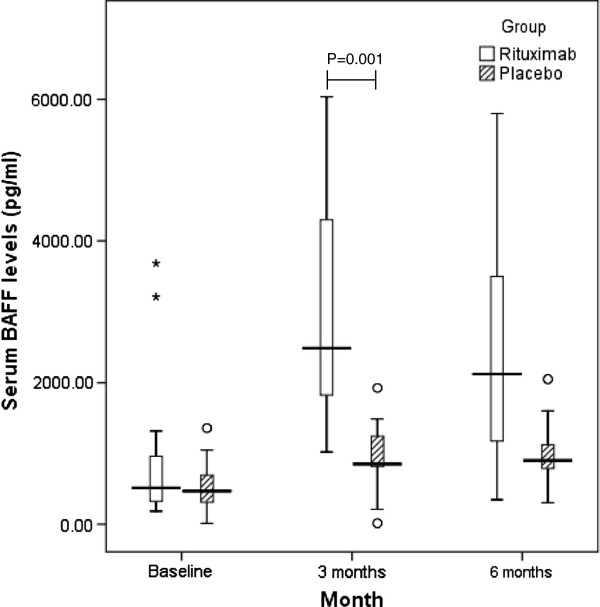
**The effects of Rituximab and Placebo on serum BAFF (ng/ml).** There was a difference in effect of Rituximab at 3 months but not at end of 6 months. (° = outliers with values more than 1.5 times the height of the box; * = outliers with values more than 3 times height of box).

Patient demographics have been published previously [8]. The mean age was 40 years and 55% of patients were females. There were no significant differences between the treatment and placebo groups for both serum IgE (reduced from a baseline median of 17.95 IU/ml (61.07, IQR) to 11.28 IU/ml (25.67, IQR) at 6 months in treatment arm compared to a baseline median of 27.53 IU /ml (89.35, IQR) to 11.97 IU/ml (47.0, IQR) at 6 months in the placebo group; mean difference between groups in log units at 6 months was 0.145, 95% CI −0.24 to 0.53, Figure 1) and BAFF (increased from a baseline median of 512.9 pg/ml (613.36, IQR) to 2119.16 pg/ml (2325.35, IQR) at 6 months in treatment arm compared to a baseline median of 468.39pg/ml (381.43, IQR) to 900.30 pg/ml (335.03, IQR) at 6 months in the placebo group; mean difference between groups in log units at 6 months was −0.3756, 95% CI −0.57 to - 0.18, Figure 2). Further, no significant reduction in serum IgE was noted at the end of 3 months or in a subgroup analysis (baseline IgE levels <60 IU/l, 60-120 IU/l and >120 IU/l). But the BAFF levels did increase significantly at the end of 3 months from a baseline median of 512.9 pg/ml (613.36, IQR) to 2487.03 pg/ml (2473.28 IQR) at 3 months in the treatment arm compared to a baseline median of 468.39pg/ml (381.43, IQR) to 850.80 pg/ml (424.28, IQR) at 3 months in the placebo group; mean difference between groups in log units −0.549, 95% CI −0.33 to - 0.77). There was no effect on total blood eosinophil counts as well (data not shown).

Our objective of this proof-of-activity-analysis was to determine if there may be a beneficial effect of rituximab on serum atopy markers, specifically IgE and BAFF. Had there been a significant effect, this would provide evidence to support a larger clinical trial of rituximab in patients with severe asthma and raised serum IgE. In contrast to our hypothesis, a difference between serum IgE and serum BAFF levels was not observed between placebo and rituximab treated patients.

There was also no sustained difference in BAFF levels between the two groups at 6 months despite there being an increment of approximately 5 times the baseline at the end of the 3^rd^ month.

This is the first randomized placebo-controlled clinical trial that has evaluated the long-term effect of rituximab on serum IgE and BAFF, despite being a post hoc analysis of a clinical trial of an alternate disease that is unrelated to atopy. Due to the design of the initial study which focussed primarily on the efficacy of the drug on platelet function, data on patient’s atopic status was not available. We also had no information on the participants medical history including asthma symptoms, airway or systemic inflammation, lung function of patients or the history of use of immunomodulators prior to the onset of the trial. Unlike many atopic patients, those in this study had IgE levels in the normal range. Therefore our study was unable to determine if there may be an effect of rituximab treatment for patients with atopic conditions who have a high baseline IgE. This apparent inability to lower serum IgE levels is likely due to plasma cells having a low expression of CD20 [9] and therefore remaining unaffected by Rituximab. (). The effect of rituximab treatment on tissue IgE stores were not assessed in this study. These tissue stores of IgE are considerable, IgE is localized in the tissue to the high affinity IgE receptor (FCepsilonR1) on mast cells and basophils. These tissue stores are unlikely to be affected by destruction of IgE producing B cells due to the long half-life of IgE bound to the high affinity IgE receptor.

The lack of significant increase in BAFF levels too is likely an indication of the fact that BAFF levels may be regulated by pathways other than B cell. BAFF is reported to be produced constitutively by stromal cells within lymphoid organs [10] and is inducible by most cells of myeloid origin (monocytes, macrophages, neutrophils, and dendritic cells).

In summary, we demonstrate that rituximab does not affect serum IgE or BAFF levels in a population of unselected subjects with varying levels of baseline serum IgE. This suggests that rituximab may not be a useful treatment option for patients with severe atopic asthma.

## Competing interest

None of the authors have any competing interests relevant to the data presented in this manuscript.

## Authors’ contribution

PN conceived the idea, DA collected the data, KR performed the biochemical analysis, ADG and LT did the statistical analysis, ADG prepared the first draft of the manuscript, PN, DA and LT critically evaluated the manuscript, PN acts as the guarantor for the manuscript. All others read and approved the final manuscript
